# Differential effects of cyclo-oxygenase 1 and 2 inhibition on angiogenesis inhibitor-induced hypertension and kidney damage

**DOI:** 10.1042/CS20220182

**Published:** 2022-05-10

**Authors:** Katrina M. Mirabito Colafella, Daan C.H. van Dorst, Rugina I. Neuman, Leni van Doorn, Karla Bianca Neves, Augusto C. Montezano, Ingrid M. Garrelds, Richard van Veghel, René de Vries, Estrellita Uijl, Marian C. Clahsen-van Groningen, Hans J. Baelde, Anton H. van den Meiracker, Rhian M. Touyz, Willy Visser, A.H. Jan Danser, Jorie Versmissen

**Affiliations:** 1 Cardiovascular Disease Program, Biomedicine Discovery Institute and Department of Physiology, Monash University, Melbourne, Australia; 2 Division of Pharmacology and Vascular Medicine, Department of Internal Medicine, Erasmus MC, University Medical Centre, Rotterdam, The Netherlands; 3 Department of Medical Oncology, Erasmus MC Cancer Institute, Rotterdam, The Netherlands; 4 British Heart Foundation Glasgow Cardiovascular Research Centre, Institute of Cardiovascular and Medical Sciences, University of Glasgow, United Kingdom; 5 Department of Pathology, Erasmus MC University Medical Centre, Rotterdam, The Netherlands; 6 Department of Pathology, Leiden University Medical Centre, Leiden, The Netherlands; 7 Department of Hospital Pharmacy, Erasmus MC University Medical Centre, Rotterdam, The Netherlands

**Keywords:** cyclooxygenase, endothelins, hypertension, NADPH oxidase, vascular endothelial growth factor

## Abstract

Vascular endothelial growth factor antagonism with angiogenesis inhibitors in cancer patients induces a ‘preeclampsia-like’ syndrome including hypertension, proteinuria and elevated endothelin (ET)-1. Cyclo-oxygenase (COX) inhibition with aspirin is known to prevent the onset of preeclampsia in high-risk patients. In the present study, we hypothesised that treatment with aspirin would prevent the development of angiogenesis inhibitor-induced hypertension and kidney damage. Our aims were to compare the effects of low-dose (COX-1 inhibition) and high-dose (dual COX-1 and COX-2 inhibition) aspirin on blood pressure, vascular function, oxidative stress, ET-1 and prostanoid levels and kidney damage during angiogenesis-inhibitor therapy in rodents. To this end, Wistar Kyoto rats were treated with vehicle, angiogenesis inhibitor (sunitinib) alone or in combination with low- or high-dose aspirin for 8 days (*n*=5–7/group). Our results demonstrated that prostacyclin (PGI_2_) and ET-1 were increased during angiogenesis-inhibitor therapy, while thromboxane (TXA_2_) was unchanged. Both low- and high-dose aspirin blunted angiogenesis inhibitor-induced hypertension and vascular superoxide production to a similar extent, whereas only high-dose aspirin prevented albuminuria. While circulating TXA_2_ and prostaglandin F_2α_ levels were reduced by both low- and high-dose aspirin, circulating and urinary levels PGI_2_ were only reduced by high-dose aspirin. Lastly, treatment with aspirin did not significantly affect ET-1 or vascular function. Collectively our findings suggest that prostanoids contribute to the development of angiogenesis inhibitor-induced hypertension and renal damage and that targeting the prostanoid pathway could be an effective strategy to mitigate the unwanted cardiovascular and renal toxicities associated with angiogenesis inhibitors.

## Introduction

Angiogenesis, the formation of new blood vessels from the pre-existing vasculature, is essential for tumour growth and metastatic spread. Inhibition of angiogenesis by targeting the vascular endothelial growth factor (VEGF) pathway, either alone or in combination with other therapies such as the latest breakthrough cancer therapy, immune checkpoint inhibitors, is an effective strategy for various types of cancer and leads to prolonged patient survival [1–3]. However, angiogenesis inhibitors can induce severe cardiovascular and renal toxicities, most frequently hypertension and nephropathy, which may require dose reduction or early termination of therapy. Four types of angiogenesis inhibitors are used clinically: anti-VEGF monoclonal antibodies, VEGF-soluble decoy receptors that capture freely available VEGF, anti-VEGF receptor (VEGFR) monoclonal antibodies and multitargeted tyrosine kinase inhibitors (TKIs) such as sunitinib that have anti-VEGFR activity. Of note, almost all patients experience a rise in blood pressure, with the incidence of hypertension ranging from 4 to 84% depending on the angiogenesis inhibitor used [1], demonstrating that this is a class effect rather than a phenomenon linked to a particular type of angiogenesis inhibitor. Understanding the mechanisms underlying angiogenesis inhibitor-induced hypertension and nephropathy is integral if we are to develop preventative strategies to protect cardiovascular health and allow cancer patients to remain on this lifesaving treatment.

Angiogenesis inhibitor-induced hypertension and kidney damage recapitulate the clinical presentation of preeclampsia, including glomerular endotheliosis, proteinuria and a rise in endothelin (ET)-1 [4]. Since preeclampsia is characterised by high levels of soluble Fms-like tyrosine kinase 1 (sFlt-1), a naturally occurring antagonist of VEGF, it is perhaps not surprising that angiogenesis inhibitors cause a ‘preeclampsia-like’ syndrome and that the underlying mechanisms are thought to be the same [1,5,6]. Here it is of interest to note that cyclo-oxygenase (COX) inhibition with aspirin can prevent the onset of preeclampsia in high-risk patients [6]. Although the initial studies used higher doses of aspirin, resulting in dual COX-1 and COX-2 inhibition [7,8], clinical trials have only investigated the efficacy of low-dose aspirin (i.e., selective COX-1 inhibition) [9,10]. Further, while the protective effect of aspirin is primarily attributed to the restoration of the prostacyclin (PGI_2_)/thromboxane (TXA_2_) ratio [11], other mechanisms such as a reduction in oxidative stress and decreased activation of the ET system are likely to contribute as well [6]. Importantly, these effects may be linked to COX-2 rather than COX-1 dependent prostanoid generation [6].

In the present study, we hypothesised that treatment with aspirin would prevent the development of angiogenesis inhibitor-induced hypertension and kidney damage. To test this hypothesis, we utilised an established model of angiogenesis inhibitor (sunitinib)-induced hypertension and kidney damage [4,12–16]. Our aims were to compare the effects of low-dose (COX-1 inhibition) and high-dose (dual COX-1 and COX-2 inhibition) aspirin on blood pressure, vascular function, oxidative stress, ET-1 and prostanoid levels and kidney damage during angiogenesis-inhibitor therapy in rodents. To this end, male Wistar Kyoto (WKY) rats were treated with vehicle, the angiogenesis inhibitor, sunitinib, alone or in combination with low- or high-dose aspirin for 8 days.

## Materials and methods

### *In vivo* study

Animal experiments were performed at the Erasmus Medical Centre (Rotterdam, The Netherlands). The experiments were approved by the Animal Ethics Committee of the Erasmus University Medical Center Rotterdam (protocol number 118-16-01) and were performed in accordance with the guidelines from Directive 2010/63/EU of the European Parliament and the Netherlands Experiments on Animals Act (Wod, 2014). Male WKY rats were obtained at 10 weeks of age from Charles River, Germany and Janvier Labs, France. Animals were housed in an experimental room with temperature maintained at 21–22°C and a 12-h light–dark cycle. Rats had *ad libitum* access to normal rodent chow and water.

Following a 1-week acclimatisation period, rats were anaesthetised using Forane (isoflurane; inhalation anaesthetic at a dose of 2–3% for induction and then maintained at 2% for the duration of the surgery) for implantation of a radiotelemetry probe (HD-S10, Data Sciences International, MN, U.S.A.) into the abdominal aorta as described previously [15]. Analgesia (temgesic, 0.05 mg/kg s.c.; RB Pharmaceuticals) was administered prior to the surgery and for 2 days afterwards. Following a 10-day recovery period, arterial pressures and heart rate were measured via radiotelemetry as described previously [16]. After establishing baseline values, rats were randomly assigned to one of the four treatment groups: vehicle (*n*=6), the angiogenesis inhibitor sunitinib (14 mg/kg/day; SU; *n*=7) alone or in combination with low-dose aspirin (5 mg/kg/day; SU+low-dose A; *n*=5) or high-dose aspirin (100 mg/kg/day; SU+high-dose A; *n*=5). The low- and high-dose aspirin correspond to human equivalent doses of ∼60 and ∼1200 mg, respectively. Previous studies have demonstrated that these doses of aspirin result in selective COX-1 inhibition and dual COX-1 and COX-2 inhibition, respectively [17–19]. Some of the data for the vehicle and SU-treated groups and a subset of the data from the SU+high-dose A treated group have been reported recently [16]. Treatments were administered by oral gavage for 8 days. Arterial pressures and heart rate were measured on days 1–6 of treatment. On days 7–8 of treatment, rats were placed into metabolic cages to collect a 24-h urine sample. Thereafter, rats were killed by Forane (isoflurane) anaesthesia overdose and exsanguinated via abdominal vein puncture. Blood was collected into a heparinised tube and then centrifuged at 3000 rpm to obtain plasma. Thereafter, plasma was stored at −80°C for later analysis.

### *Ex vivo* vascular function

Vascular function was assessed in response to the vasodilator acetylcholine (ACh) or the vasoconstrictor ET-1 in isolated iliac segments as described previously [16]. In additional segments, ACh experiments were performed in the presence (30 min pre-incubation) of NO-synthase inhibition with l-ω-Nitro-l-arginine methyl ester hydrochloride (l-NAME; 100 μmol/l), endothelium-derived hyperpolarising factor inhibition with the intermediate conductance Ca^2+^-activated K^+^ channel inhibitor TRAM34 (10 μmol/l) or the small conductance Ca^2+^-activated K^+^ channel inhibitor apamin (100 μmol/l) or their combination and for ET-1, in the presence of ET_A_ receptor (BQ123; 1 μmol/l) or ET_B_ receptor (BQ788; 1 μmol/l) blockade as described previously [16].

### Circulating and urinary measurements

ELISAs were used to determine circulating and urinary ET-1 (ET-1 Quantikine ELISA Kit DET100, R&D systems), PGF_2α_ (circulating PGF_2α_ via 8-isoprostane ELISA Kit No. 516351, Cayman Chemical and urinary PGF_2α_ via Direct 8-iso-PGF_2α_ ELISA Kit ADI-900-091, Enzo Life Sciences), PGI_2_ (6-keto-PGF_1α_ ELISA Kit ADI-900-004, Enzo Life Sciences) and TXA_2_ (TXB_2_ ELISA Kit ADI-900-002, Enzo Life Sciences) levels and the urinary excretion of albumin (Rat ELISA Ab108790, Abcam), neutrophil gelatinase-associated lipocalin (NGAL; Rat lipocalin-2 ELISA Kit Ab207925, Abcam), kidney injury molecule-1 (KIM-1) (Rat TIM-1/KIM-1/HAVCR Quantikine ELISA Kit RKM100, R&D Systems), PGE_2_ (Prostaglandin E_2_ ELISA Kit No. 514010, Cayman Chemical) and its metabolite, PGE-M (Prostaglandin E Metabolite ELISA Kit No. 514531, Cayman Chemical).

### Markers of oxidative stress

To assess oxidative stress, aortic and renal superoxide anion (O_2_^−^) levels were measured by lucigenin chemiluminescence assay as described previously [20]. Aortic, cardiac and renal hydrogen peroxide (H_2_O_2_) levels were measured by Amplex Red assay (A22188; Life Technologies, Paisley, U.K.). Aortic mRNA expression of NADPH oxidase (Nox) isoforms 1, 2 and 4 and antioxidant enzymes (SOD1, catalase, GXP1, HO1, Trdx1 and Prdx1) were determined by qPCR using SYBR Green PCR Master Mix (Applied Biosystems, U.K.) and the relative mRNA expression (target gene/18S housekeeping gene) was calculated using the ΔΔ*C*_t_ method as described previously [16]. RNA was isolated from the renal cortex using TRIzol (Ambion, Foster City, CA), in accordance with the manufacturer’s instructions and reverse transcribed into cDNA using an AMV cDNA synthesis kit (Roche, Indianapolis, IN). Primers used are as described previously [16]. Aortic protein expression of Nox 1, 2 and 4 were determined using Western blotting. Protein (30 µg) was separated by electrophoresis on a polyacrylamide gel and transferred to a nitrocellulose membrane. Non-specific-binding sites were blocked with 3% bovine serum albumin in Tris-buffered saline (TBS) solution. Membranes were then incubated with specific antibodies overnight at 4°C. Membranes were washed three times with TBS-Tween20 and incubated with infrared dye-labelled secondary antibodies for 1 h at room temperature. Results were visualised using an Odyssey CLx infrared imaging system (Li-COR Biosciences U.K. Ltd, U.K.) and results were normalised to α-tubulin protein and are expressed in arbitrary units compared with vehicle group. Antibodies used were as follows: anti-α-tubulin (1:5000; Abcam, U.K.); anti-Nox1 (1:1000; Sigma, U.K.); anti-Nox2 (1:1000; Abcam, U.K.); anti-Nox4 (1:500; Santa Cruz, U.K.).

### Renal mRNA and protein expression

Renal mRNA expression of nephrin, podocin, VEGF, ECE, ET-1, ET_A_ receptor, ET_B_ receptor, COX-1, COX-2, PGI_2_ synthase and TXA_2_ synthase were determined via qPCR using iQSYBR Green supermix (Bio-Rad) and the relative mRNA expression (target gene/hypoxanthine phosphoribosyltrasferase-1 (*Hprt1*) housekeeping gene) was calculated using the ΔΔ*C*_t_ method as described previously [16]. RNA was isolated from the renal cortex using TRIzol (Ambion, Foster City, CA), in accordance with the manufacturer’s instructions and reverse transcribed into cDNA using an AMV cDNA synthesis kit (Roche, Indianapolis, IN). Primers used are as described previously [16]. Renal protein abundance of the ET_A_ and ET_B_ receptors were determined using Western blotting. Protein (40 µg) was separated by electrophoresis on a polyacrylamide gel and transferred to a nitrocellulose membrane. Non-specific-binding sites were blocked with 5% bovine serum albumin in TBS solution containing 0.1% Tween-20. Membranes were then incubated with specific antibodies overnight at 4°C. Membranes were washed three times with TBS-Tween20 and incubated with infrared dye-labelled secondary antibodies for 1 h at room temperature. Signals were detected by chemiluminescence (Clarity Western ECL substrate; Bio-Rad) and quantified using Image Studio Lite software. Results were normalised to β-actin protein and are expressed in arbitrary units compared with vehicle group. Antibodies used were as follows: anti-ET_A_ (1:1000, Abcam, U.K.), anti-ET_B_ (1:1000, Abcam, U.K.), β-actin (1:1000, Cell Signaling 4967).

### Qualitative assessment of kidney morphology

Transverse kidney samples were fixed in 3.5–4% formaldehyde solution for light microscopy evaluation. After fixation, tissues were dehydrated, and paraffin embedded. Deparaffinised 2-μm-thick sections were stained for Periodic Acid–Schiff (PAS) as described previously. In PAS-stained sections, the glomeruli were blindly evaluated by a pathologist (Marian C. Clahsen-van Groningen) for the presence or absence of endothelial cell and epithelial cell swelling and the presence of ischaemia and intraepithelial protein. Kidney biopsies from vehicle and sunitinib-treated animals (*n*=2/group) were immersed in Karnovsy’s fixative containing 2% glutaraldehyde in 0.1 M sodium cacodylate buffer and processed for transmission electron microscopy as described previously [14]. Glomeruli were blindly evaluated by a pathologist (Marian C. Clahsen-van Groningen) for the occurrence of glomerular endotheliosis (endothelial cell swelling, encroachment of the capillary spaces and loss of endothelial fenestrations) and podocyte morphology.

### Statistical analyses

Data are presented as mean ± SEM and were analysed using a one-way ANOVA followed by post-hoc *t* tests with Holm–Sidak correction to reduce the risk of type-1 error associated with multiple comparisons. *P*≤0.05 was considered to be statistically significant.

## Results

### Low- and high-dose aspirin blunted sunitinib-induced hypertension to a similar extent

**Figure 1:**
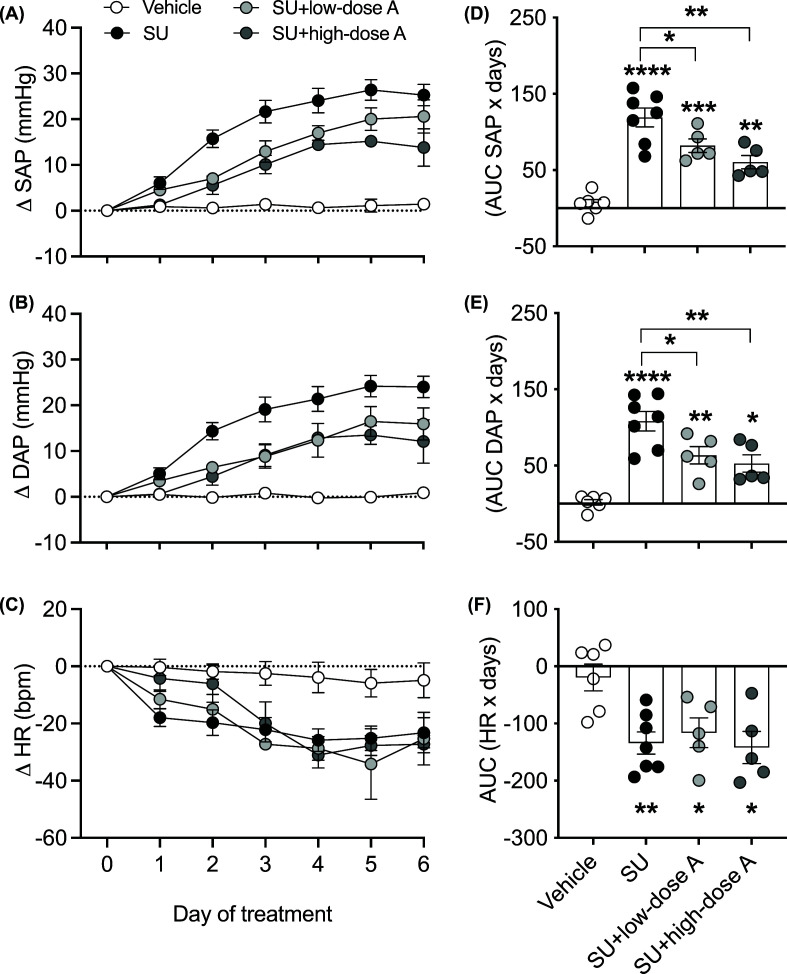
The effect of treatment on blood pressures and heart rate Changes in systolic arterial pressure (SAP), diastolic arterial pressure (DAP) and heart rate (HR) in response to treatment with vehicle, sunitinib (14 mg/kg/day) alone or during co-treatment with low-dose aspirin (COX-1 inhibition, 5 mg/kg/day; SU+low-dose A) or high-dose aspirin (dual COX-1 and COX-2 inhibition, 100 mg/kg/day; SU+high-dose A). Time course of changes in (**A**) SAP, (**B**) DAP and (**C**) HR and areas under the curve (AUCs) for the cumulative change in (**D**) SAP, (**E**) DAP and (**F**) HR. Data are presented as mean ± SEM (*n*=5–7/group). Data were analysed by one-way ANOVA followed by Holm–Sidak’s post-hoc test. *P<0.5, **P<0.01, ***P<0.001, ****P<0.0001 versus vehicle-treated unless otherwise indicated.

Basal systolic and diastolic arterial pressures and heart rate were 135 ± 1 mmHg, 90 ± 1 mmHg and 333 ± 5 bpm, respectively. Sunitinib induced a rapid pressor response, with the increase in systolic and diastolic arterial pressures measuring 25 ± 2 and 24 ± 2 mmHg, respectively, on day 6 of treatment (Figure 1A,B). Low-dose aspirin blunted the systolic pressor response to sunitinib by 31% (*P*<0.05 versus sunitinib alone; Figure 1A,D). This effect was slightly greater during co-treatment with high-dose aspirin, such that the rise in systolic arterial pressure in response to sunitinib was attenuated by 49% (*P*<0.01 versus sunitinib alone, *P*=0.16 versus co-treatment with low-dose aspirin; Figure 1A,D). Similar changes were observed in diastolic arterial pressure during co-treatment with low- and high-dose aspirin (Figure 1B,E). Sunitinib significantly reduced heart rate (*P*<0.01 versus vehicle-treated) and this response was unaffected by co-treatment with low- or high-dose aspirin (Figure 1C,F).

**Figure 2:**
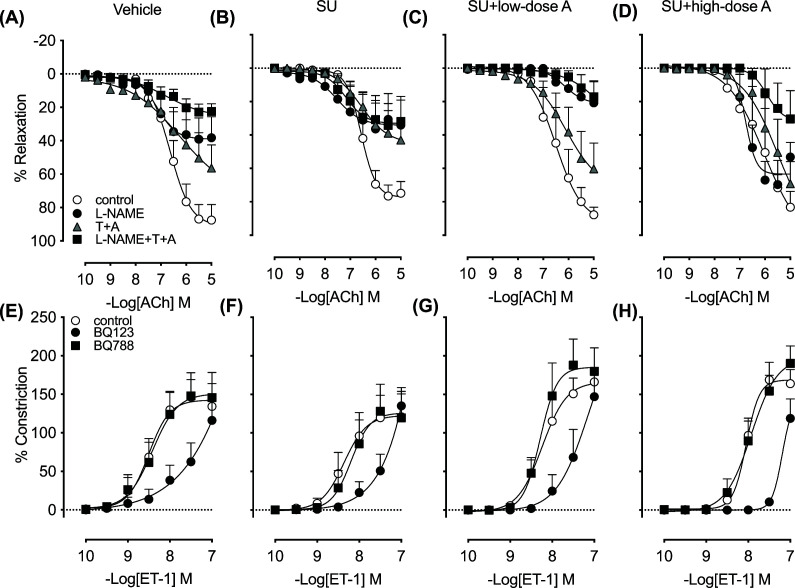
The effect of treatment on vascular function Iliac artery concentration–response curves to (**A–D**) ACh in the absence or presence of l-NAME (100 μmol/l), TRAM34 (10 μmol/l) and apamin (100 μmol/l) (T+A) or their combination (l-NAME+T+A) and for (**E–H**) ET-1, in the absence or presence of BQ123 (1 μmol/l) or BQ788 (1 μmol/l) following 8 days of treatment with vehicle, sunitinib (14 mg/kg/day; SU) alone or in combination with low-dose aspirin (COX-1 inhibition, 5 mg/kg/day; SU+low-dose A) or high-dose aspirin (dual COX-1 and COX-2 inhibition, 100 mg/kg/day; SU+high-dose A) in WKY rats. Data are presented as mean ± SEM (*n*=4–7/group). Relaxation data are expressed as a percentage of the response to U46619 (0.1–0.3 μmol/l). See Table 1 for statistical information.

To determine whether the effect of aspirin on the pressor response to sunitinib was associated with altered vascular reactivity to ACh and ET-1, concentration–response curves were constructed *ex vivo* in isolated iliac arteries. The vasodilator response to ACh was comparable between the vehicle and sunitinib-treated groups and was unaffected by co-treatment with low- or high-dose aspirin (Figure 2A–D and Table 1). Similarly, in the presence of NOS inhibition (with l-NAME) alone or in combination with endothelium-derived hyperpolarising factor inhibition (with TRAM34 and apamin), the vasodilator response to ACh was reduced by a similar extent between the groups, except for sunitinib group co-treated with high-dose aspirin (Figure 2A–D and Table 1). In the sunitinib only treated group, the vasodilator response to ACh tended to be reduced in the presence of TRAM34 and apamin (*P*=0.07 versus control segment; Figure 2B and Table 1). The magnitude of the vasoconstrictor response to ET-1 was similar between the groups and was not significantly altered by ET_A_ receptor blockade (with BQ123) or ET_B_ receptor blockade (with BQ788) (Figure 2E–H and Table 1). However, in the presence of BQ123 the sensitivity to ET-1 was reduced in all groups such that the curve was shifted rightward (Figure 2E–H and Table 1).

**Table 1:** pEC_50_ and *E*_max_ values for iliac artery concentration–response curves for ACh in the absence or presence of l-NAME (100 μmol/l), TRAM34 (10 μmol/l) and apamin (100 μmol/l) (T+A) or their combination (l-NAME+T+A) and ET-1 in the absence or presence of BQ123 (1 μmol/l) or BQ788 1 (μmol/l) following 8 days of treatment with vehicle, sunitinib (14 mg/kg/day) alone or in combination with low-dose aspirin (5 mg/kg/day; SU+low-dose A) or high-dose aspirin (100 mg/kg/day; SU+high-dose A) in WKY rats

	Vehicle	SU	SU+low-dose A	SU+high-dose A
**pEC_50_**				
ACh	6.7 ± 0.2	6.6 ± 0.1	6.5 ± 0.3	6.3 ± 0.4
ACh+l-NAME	6.9 ± 0.4	7.7 ± 0.6	6.4 ± 0.4	6.6 ± 0.2
ACh+T+A	6.6 ± 0.4	6.7 ± 0.3	6.3 ± 0.4	5.9 ± 0.3
ACh+l-NAME+T+A	7.0 ± 0.5	7.4 ± 0.3	6.4 ± 0.5	6.5 ± 0.4
ET-1	8.5 ± 0.1	8.3 ± 0.2	8.2 ± 0.2	8.0 ± 0.1
ET-1+BQ123	7.5 ± 0.2^§^	7.5 ± 0.2^‡^	7.4 ± 0.2^†^	7.3± 0.1^†^
ET-1+BQ788	8.4 ± 0.1	8.2 ± 0.1	8.2 ± 0.1	7.9 ± 0.1
***E*_max_**				
ACh	89 ± 10	77 ± 6	90 ± 4	84 ± 10
ACh+l-NAME	39 ± 13*	36 ± 16*	28 ± 14^†^	65 ± 6
ACh+T+A	65 ± 18	44 ± 13	80 ± 21	75 ± 12
ACh+l-NAME+T+A	24 ± 4^†^	37 ± 13*	20 ± 12^†^	30 ± 17*
ET-1	145 ± 23	132 ± 31	165 ± 14	172 ± 18
ET-1+BQ123	148 ± 37	159 ± 31	202 ± 46	150 ± 37
ET-1+BQ788	149 ± 31	125 ± 35	189 ± 30	200 ± 28

Data are presented as mean ± SEM (*n*=4–7/group). *P<0.5. ^†^*P*<0.01. ^‡^*P*<0.001. ^§^*P*<0.0001, ^‡^*P*<0.001, ^†^*P*<0.01 versus control segment.

### Effect of low- and high-dose aspirin on circulating ET-1 and prostanoid levels during sunitinib treatment

**Figure 3:**
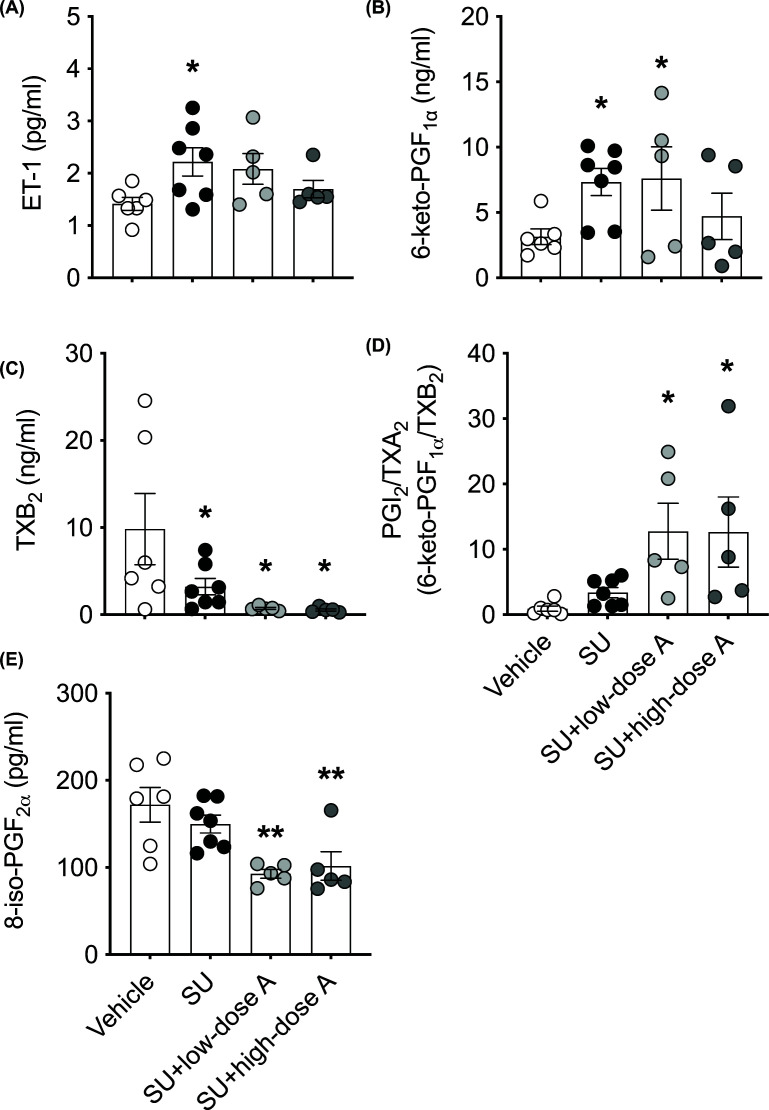
The effect of treatment on circulating endothelin (ET)-1 and prostanoid levels Circulating levels of (**A**) ET-1 and (**B**) PGI_2_, (**C**) TXA_2_, (**D**) the PGI_2_/TXA_2_ ratio and (**E**) PGF_2α_ as measured by their metabolites, 6-keto-PGF_1α_, TXB_2_, and 8-iso-PGF_2α_ respectively, following treatment with vehicle, sunitinib (14 mg/kg/day; SU) alone or during co-treatment with low-dose aspirin (COX-1 inhibition, 5 mg/kg/day; SU+low-dose A) or high-dose aspirin (dual COX-1 and COX-2 inhibition, 100 mg/kg/day; SU+high-dose A). Data are presented as mean ± SEM (*n*=5–7/group). Data were analysed using a one-way ANOVA followed by post-hoc *t* tests with Holm–Sidak correction where appropriate. *P≤0.05, **P<0.01 versus vehicle-treated.

Circulating ET-1 was higher in the sunitinib-treated groups, however this only reached statistical significance when sunitinib was administered alone (*P*=0.05 versus vehicle-treated; Figure 3A). PGI_2_, measured via its stable metabolite 6-keto-PGF_1α_, was increased during treatment with sunitinib alone and this response was unaffected by co-treatment with low-dose aspirin (both *P*<0.05 versus vehicle-treated; Figure 3B) and reduced by 30% during co-treatment with high-dose aspirin (Figure 3B). Circulating TXB_2_, the stable metabolite of TXA_2_, was reduced by 67% during treatment with sunitinib and by 93 and 94% during co-treatment with low- and high-dose aspirin respectively (all *P*<0.05 versus vehicle-treated; Figure 3C). Consequently, the PGI_2_/TXA_2_ ratio was greater during treatment with sunitinib and this reached statistical significance during co-treatment with aspirin (Figure 3D). Circulating PGF_2α_, measured as 8-iso-PGF_2α_, was similar between the vehicle and sunitinib-treated groups and reduced by a similar extent during co-treatment with low- and high-dose aspirin (both *P*<0.01 versus vehicle-treated; Figure 3E).

### Both low- and high-dose aspirin prevented sunitinib-induced vascular reactive oxygen species generation

**Figure 4:**
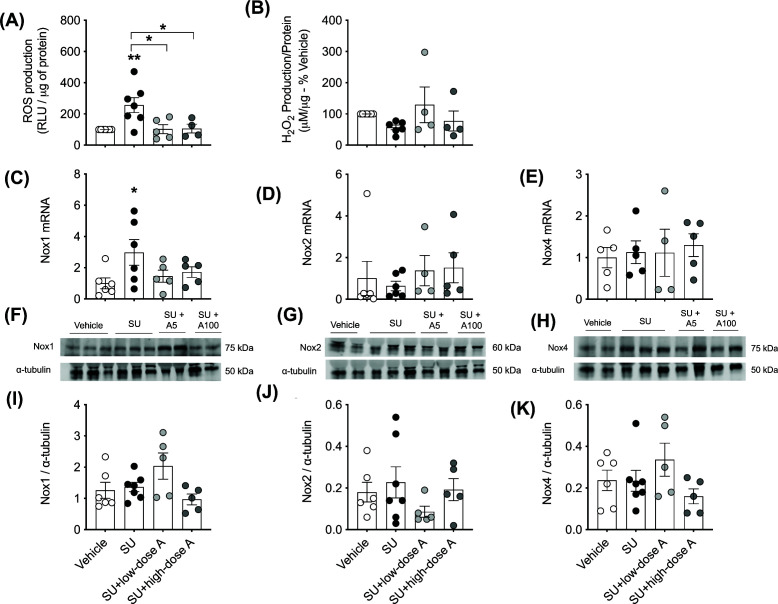
The effect of treatment on vascular oxidative stress Aortic (**A**) superoxide (O_2_^−^) generation and (**B**) H_2_O_2_ production, the mRNA expression of the pro-oxidant Nox isoforms (**C**) Nox1, (**D**) Nox2 and (**E**) Nox4 and Western blot of (**F**) Nox1, (**G**) Nox2 and (**H**) Nox4 and (**I–K**) their quantification relative to α-tubulin, respectively, following treatment with vehicle, sunitinib (14 mg/kg/day; SU) alone or during co-treatment with low-dose aspirin (COX-1 inhibition, 5 mg/kg/day; SU+low-dose A) or high-dose aspirin (dual COX-1 and COX-2 inhibition, 100 mg/kg/day; SU+high-dose A). Aortic mRNA expression is normalised to the internal housekeeping gene 18S and are expressed relative to the vehicle-treated group. Data are presented as mean ± SEM (*n*=4–7/group). Data were analysed using a one-way ANOVA followed by post-hoc *t* tests with Holm–Sidak’s correction where appropriate. *P<0.05, **P<0.01 versus vehicle-treated unless otherwise indicated.

**Figure 5:**
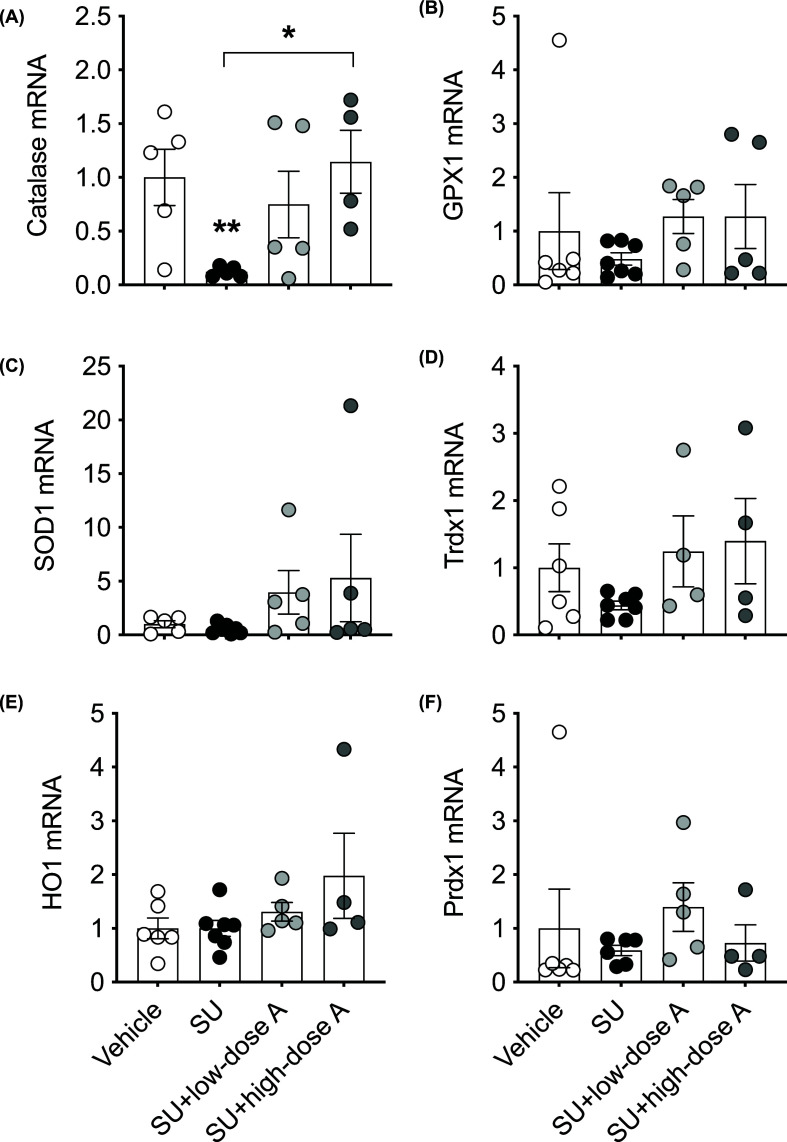
The effect of treatment on the vascular expression of antioxidants Aortic mRNA expression of the antioxidant systems (**A**) catalase (**B**) SOD1, (**C**) HO1, (**D**) GPX1, (**E**) Trdx1 and (**F**) Prdx1 following treatment with vehicle, sunitinib (14 mg/kg/day; SU) alone or during co-treatment with low-dose aspirin (COX-1 inhibition, 5 mg/kg/day; SU+low-dose A) or high-dose aspirin (dual COX-1 and COX-2 inhibition, 100 mg/kg/day; SU+high-dose A). Aortic mRNA expression is normalised to the internal housekeeping gene 18S and are expressed relative to the vehicle-treated group. Data are presented as mean ± SEM (*n*=4–7/group). Data were analysed using a one-way ANOVA. *P<0.05, **P<0.01 versus vehicle-treated unless otherwise indicated.

**Figure 6:**
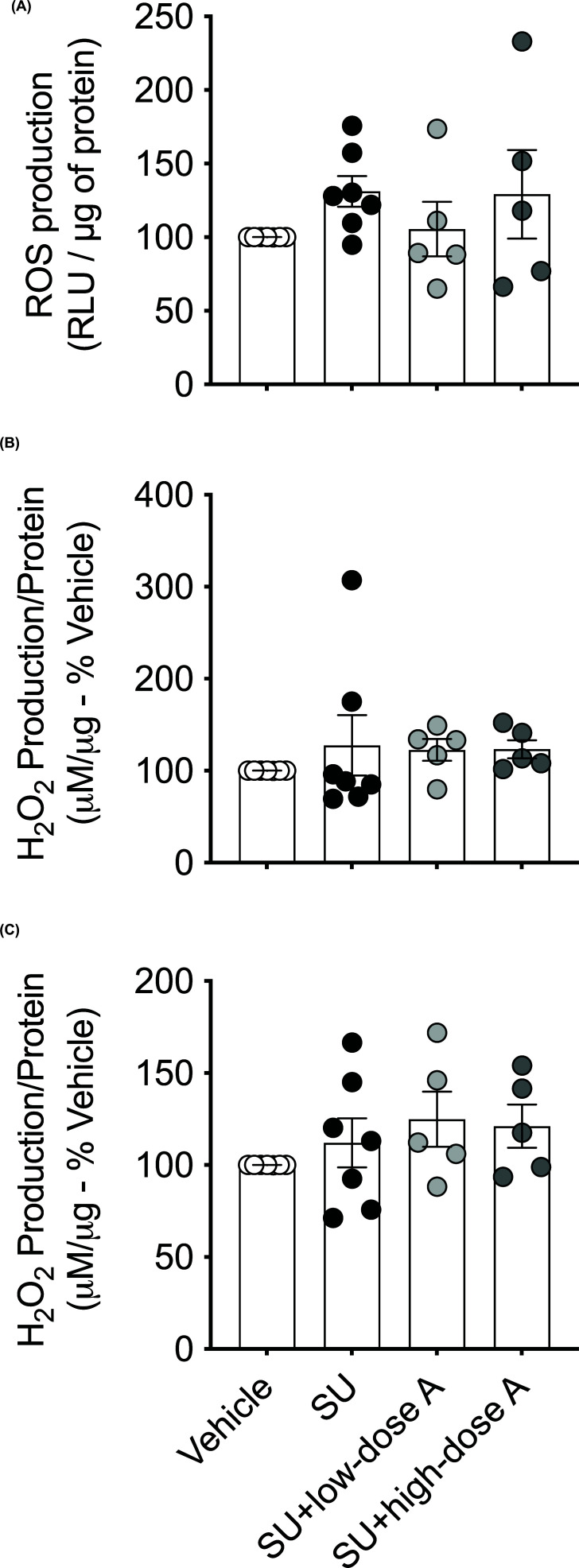
The effect of treatment on renal and cardiac oxidative stress Renal (**A**) superoxide (O_2_^−^) generation and (**B**) renal and (**C**) cardiac H_2_O_2_ production following treatment with vehicle, sunitinib (14 mg/kg/day; SU) alone or during co-treatment with low-dose aspirin (COX-1 inhibition, 5 mg/kg/day; SU+low-dose A) or high-dose aspirin (dual COX-1 and COX-2 inhibition, 100 mg/kg/day; SU+high-dose A) in WKY rats. Data are presented as mean ± SEM (*n*=5–7/group). Data were analysed using a one-way ANOVA.

Sunitinib increased aortic O_2_^−^ production (*P*=0.01 versus vehicle-treated; Figure 4A) and this effect was prevented by co-treatment with low- and high-dose aspirin (both *P*<0.05 versus sunitinib alone; Figure 4A). Conversely, aortic H_2_O_2_ levels were unaffected by treatment (Figure 4B). Aortic mRNA expression of Nox1, which is involved in the production of O_2_^−^, was higher in the sunitinib-treated group (*P*=0.02 versus vehicle-treated; Figure 4C) and this effect was absent during co-treatment with low- and high-dose aspirin (Figure 4C). There were no significant differences in the aortic mRNA expression of Nox2 and Nox4 (Figure 4D,E). While Nox1 was the most abundant Nox protein expressed, the aortic protein expression of Nox1, Nox2 and Nox 4 was similar between the groups (Figure 4F–K). However, aortic mRNA expression of catalase, which is an H_2_O_2_ scavenger was reduced by sunitinib treatment (*P*=0.002; versus vehicle-treated; Figure 5A) and this effect was prevented by high-dose aspirin (*P*=0.04 versus sunitinib alone; Figure 5A). Aortic mRNA expression for the antioxidants, SOD1, GPX1, HO1, Prdx or Trdx was similar between the treatment groups (Figure 5B–F). Renal O_2_^−^ production and renal and cardiac H_2_O_2_ levels were unaffected by treatment (Figure 6A–C).

### High-dose aspirin, but not low-dose aspirin, prevented the sunitinib-induced increase in albuminuria in association with a reduction in the urinary excretion of 6-keto-PGF_1α_

**Figure 7:**
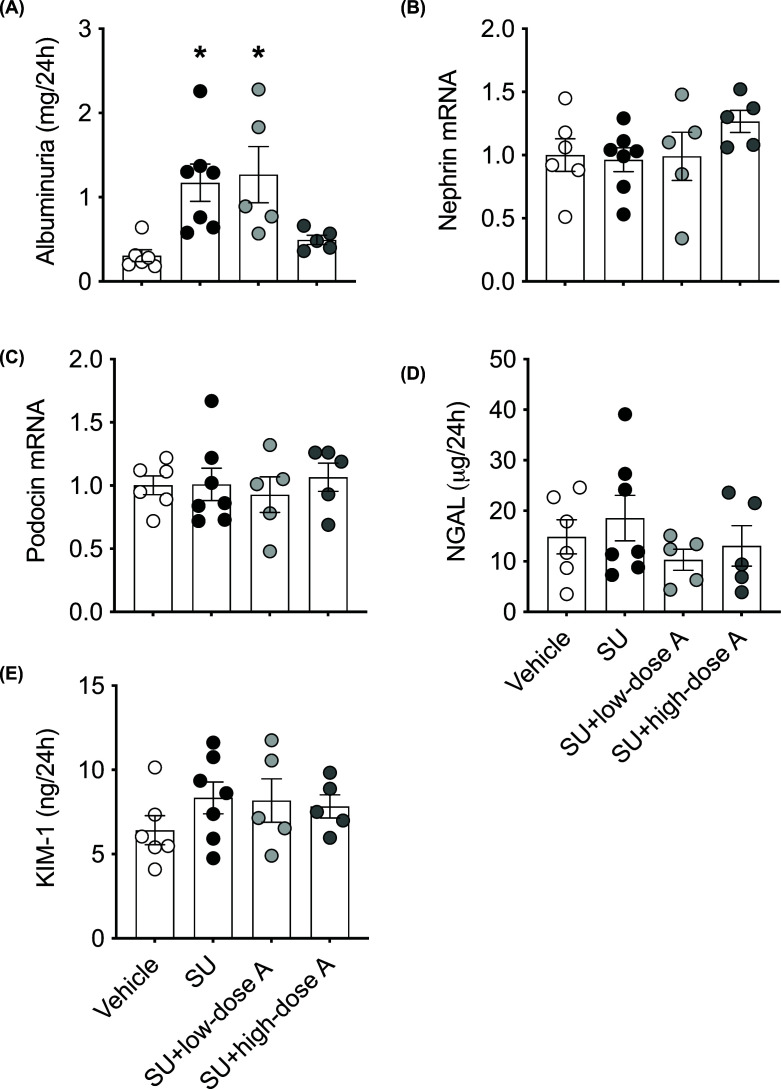
The effect of treatment on renal injury Markers of renal injury following treatment with vehicle, sunitinib (14 mg/kg/day; SU) alone or during co-treatment with low-dose aspirin (COX-1 inhibition, 5 mg/kg/day; SU+low-dose A) or high-dose aspirin (dual COX-1 and COX-2 inhibition, 100 mg/kg/day; SU+high-dose A) in WKY rats. (**A**) Albuminuria, the renal mRNA expression of (**B**) nephrin and (**C**) podocin and the urinary excretion of (**D**) NGAL and (**E**) KIM-1. Renal mRNA expression is normalised to the internal housekeeping gene, *Hprt1*, and is expressed relative to the vehicle-treated group. Data are presented as mean ± SEM (*n*=5–7/group). Data were analysed using a one-way ANOVA. *P<0.05 versus vehicle-treated.

Sunitinib treatment increased albuminuria and this effect was prevented by co-treatment with high-, but not low-dose aspirin (Figure 7A). Neither the renal mRNA expression of the genes encoding nephrin and podocin, which are essential for the normal functioning of the glomerular filtration barrier, nor the urinary excretion of NGAL and KIM-1, which are markers of tubular damage, were affected by treatment (Figure 7B–E). Qualitative light microscopy evaluation in PAS-stained kidney sections did not suggest any differences in glomerular morphology among the four groups (Supplementary Figure S1A–D). In vehicle and SU-treated rats, qualitative electron microscopy examination revealed mild loss of endothelial fenestrations and endothelial activation in response to SU treatment (Supplementary Figure S2A–D). Glomerular basement membrane thickness remained unaffected by SU treatment compared with vehicle (180 versus 164 nm, respectively).

**Figure 8:**
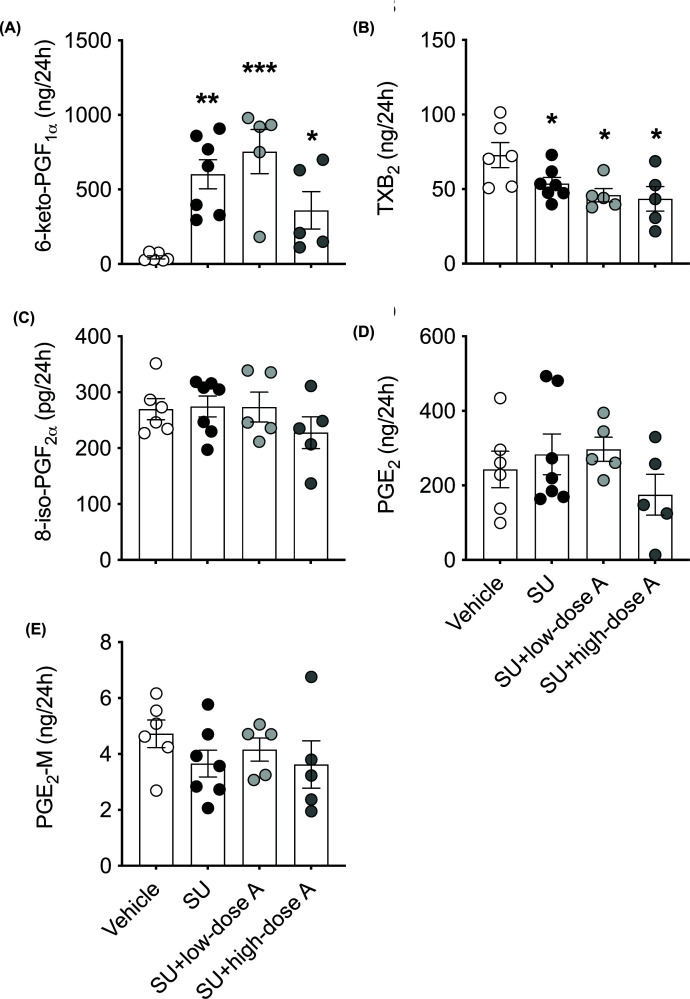
The effect of treatment on urinary prostanoid levels The urinary excretion of (**A**) PGI_2_, (**B**) TXA_2_ and (**C**) PGF_2α_ as measured by their metabolites, 6-keto-PGF_1α_, TXB_2_, and 8-iso-PGF_2α_, respectively, and (**D**) PGE_2_ and (**E**) its metabolite, PGE-M, following treatment with vehicle, sunitinib (14 mg/kg/day; SU) alone or during co-treatment with low-dose aspirin (COX-1 inhibition, 5 mg/kg/day; SU+low-dose A) or high-dose aspirin (dual COX-1 and COX-2 inhibition, 100 mg/kg/day; SU+high-dose A) in WKY rats. Data are presented as mean ± SEM (*n*=5–7/group). Data were analysed using a one-way ANOVA followed by post-hoc *t* tests with Holm–Sidak’s correction where appropriate. *P<0.05, **P<0.01, ***P<0.001 versus vehicle-treated.

**Figure 9:**
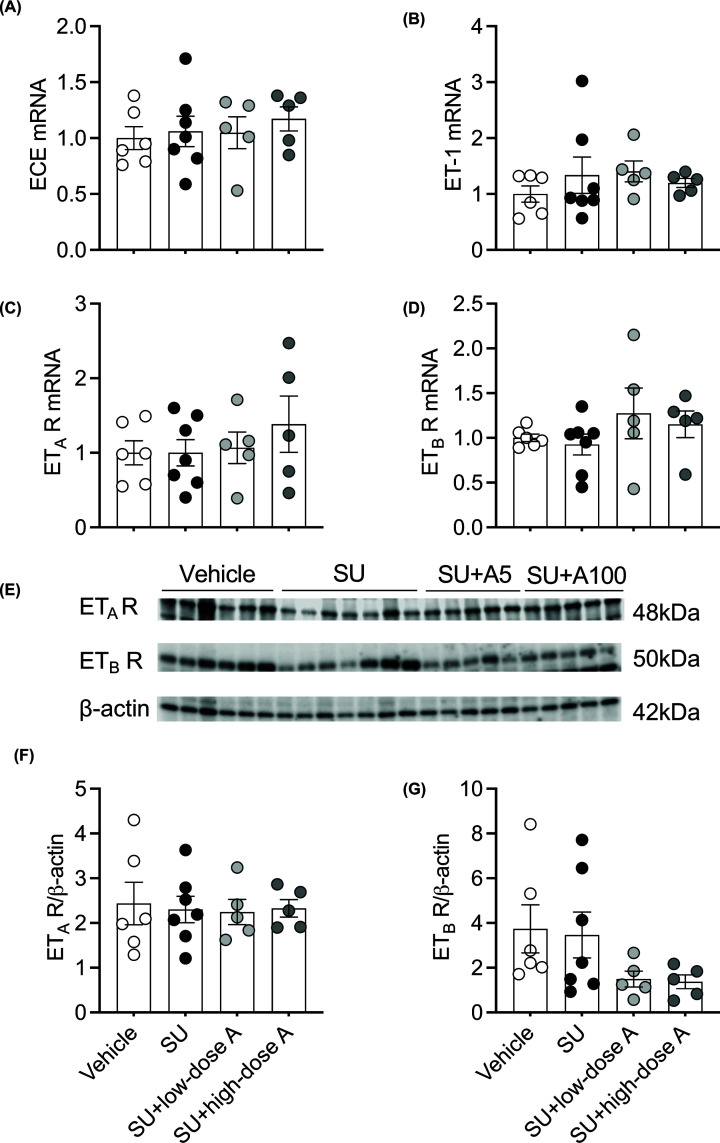
The effect of treatment on renal expression of components of the endothelin (ET) system Renal mRNA expression of (**A**) endothelial-converting enzyme (ECE), (**B**) ET-1, the ET receptors, (**C**) ET_A_ receptor (ET_A_ R) and (**D**) ET_B_ receptor (ET_B_ R), and (**E**) Western blot of ET_A_ R and ET_B_ R and (**F–G**) their quantification relative to β-actin, respectively, following treatment with vehicle, sunitinib (14 mg/kg/day; SU) alone or during co-treatment with low-dose aspirin (COX-1 inhibition, 5 mg/kg/day; SU+low-dose A) or high-dose aspirin (dual COX-1 and COX-2 inhibition, 100 mg/kg/day; SU+high-dose A). Renal mRNA expression is normalised to the internal housekeeping gene, *Hprt1*, and is expressed relative to the vehicle-treated group. Data are presented as mean ± SEM (*n*=5–7/group). Data were analysed using a one-way ANOVA.

The urinary excretion of 6-keto-PGF_1α_ was 12-fold higher following treatment with sunitinib alone, 15-fold during co-treatment with low-dose aspirin and 7-fold during co-treatment with high-dose aspirin compared with vehicle-treated rats (all *P*<0.05; Figure 8A). The urinary excretion of TXB_2_ was reduced during treatment with sunitinib alone and during co-treatment with aspirin (all *P*<0.05 versus vehicle-treated; Figure 8B). Conversely, the urinary excretion of 8-iso-PGF_2α_, PGE_2_ and PGE-M were unaffected by treatment (Figure 8C–E). Renal mRNA expression of the genes encoding VEGF, COX-1, COX-2, PGI_2_ synthase and TXA_2_ synthase were unaffected by treatment (Supplementary Figure S3A–E). Similarly, renal activation of the ET system, as assessed by mRNA expression of the genes encoding ECE, ET-1, ET_A_ receptor and ET_B_ receptor and the protein expression of the ET_A_ and ET_B_ receptors were unaffected by treatment (Figure 9A–G).

## Discussion

**Figure 10:**
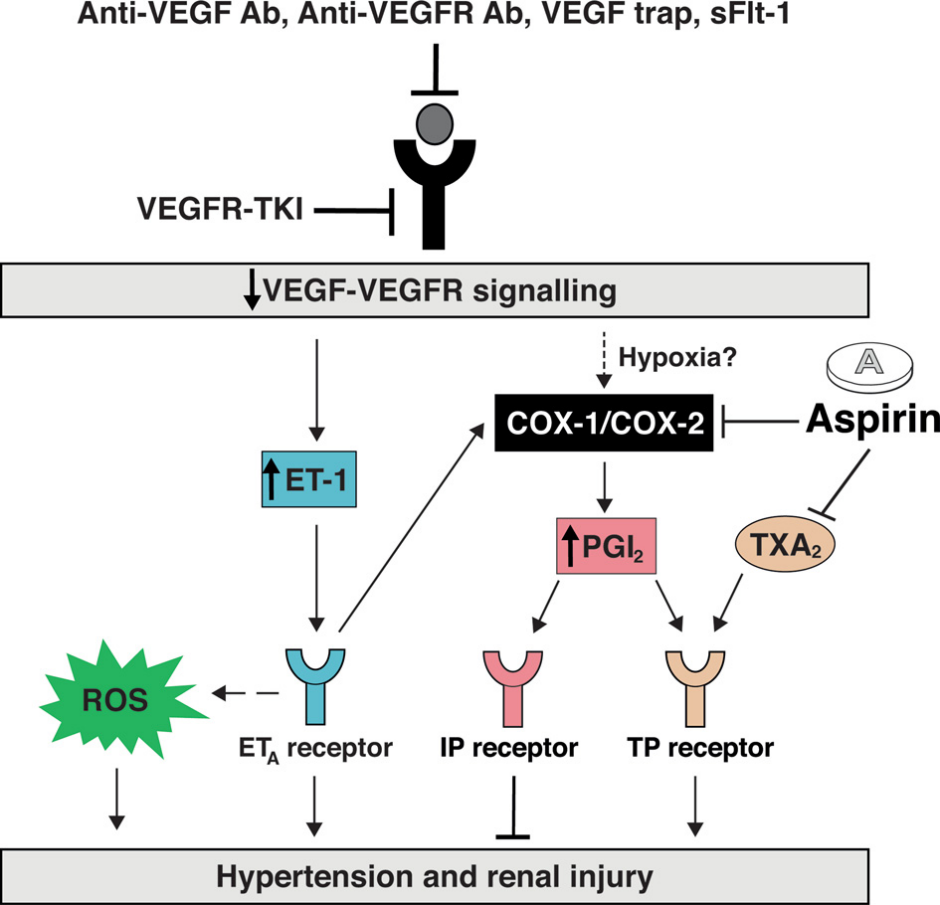
Working hypothesis of the role of the prostanoid pathway in angiogenesis inhibitor-induced hypertension and renal injury Treatment with angiogenesis inhibitors (VEGF antibodies (Abs), VEGFR Ab, VEGF trap, or TKIs), as well as elevated levels of sFlt-1 (a soluble VEGFR) in preeclampsia all lead to disturbed VEGF signaling, thereby increasing ET-1. This causes hypertension and renal injury via endothelin type A (ET_A_) receptor stimulation. Such stimulation is accompanied by reactive oxygen species (ROS) formation and COX type 1 and type 2 (COX-1/COX-2)-mediated PGI_2_ production. Normally, binding of PGI_2_ to its prostacyclin (IP) receptor results in beneficial vascular and renal effects, and thus initially this may be protective. Yet, in case of excessive PGI_2_ production, PGI_2_ can additionally stimulate other prostanoid receptors such as the thromboxane (TP) receptor, the main signaling receptor for TXA_2_, acting as an endothelium-derived contracting factor (EDCF). This could contribute to angiogenesis inhibitor-induced hypertension and nephrotoxicity. The present study has found that low-dose aspirin (resulting in selective COX-1 inhibition and TXA_2_ suppression) ameliorates TKI-induced hypertension, while high-dose aspirin (additionally resulting in COX-2 inhibition and PGI_2_ suppression) prevents renal injury.

The main findings of the present study were: (i) angiogenesis inhibitor-induced hypertension and renal injury is associated with an up-regulation in circulating and urinary PGI_2_ levels while TXA_2_, PGF_2α_ and PGE_2_ levels remained unchanged or decreased (as reported previously [16]), (ii) both low- and high-dose aspirin blunted angiogenesis inhibitor-induced hypertension and vascular superoxide production to a similar extent, whereas only high-dose aspirin prevented albuminuria, (iii) while circulating TXA_2_ and PGF_2α_ levels were reduced by both low- and high-dose aspirin, circulating and urinary levels of PGI_2_ were only reduced by high-dose aspirin and (iv) treatment with aspirin did not significantly affect ET-1 or vascular function. Collectively our findings suggest that prostanoids, and in particular PGI_2_, contribute the development of angiogenesis inhibitor-induced hypertension and renal damage and that targeting the prostanoid pathway could be an effective strategy to mitigate the unwanted cardiovascular and renal toxicities associated with angiogenesis inhibitors (Figure 10).

Since VEGF stimulates the production of PGI_2_, angiogenesis-inhibitor therapy is thought to lead to a reduction in PGI_2_, thereby contributing to the rise in blood pressure and increased thrombotic tendency [21]. However, this is yet to be confirmed in patients. Our data in an established rodent model of angiogenesis inhibitor-induced hypertension and renal injury argues against this concept [16]. The up-regulation in PGI_2_ during angiogenesis-inhibitor therapy likely occurs as a protective mechanism. The concentration of PGI_2_, which is dependent on the abundance of COX-1, COX-2 and PGI_2_ synthase, is integral in determining its effects on vascular tone and renal function. At physiological concentrations, PGI_2_ stimulates its own receptor, the prostacyclin prostanoid (IP) receptor, to elicit vasodilation and reductions in vascular remodelling, inflammation and thrombosis. However, IP receptors are desensitised and internalised after activation [22,23] and at higher concentrations, IP receptors become saturated. In this situation PGI_2_ may activate other prostanoid receptors to induce IP-like effects via the stimulation of prostaglandin E prostanoid (EP) 2, EP4 and prostaglandin D prostanoid (DP) receptors or to functionally opposing effects via stimulation of thromboxane prostanoid (TP), prostaglandin F prostanoid (FP), EP1 or EP3 receptors. Since the sensitivity of PGI_2_ is greater for TP receptors than FP, EP1 and EP3 receptors [24], it is likely that high levels of PGI_2_ during angiogenesis-inhibitor therapy primarily lead to the stimulation of TP receptors. In agreement with this hypothesis, it has been demonstrated in mice that sFlt-1-induced hypertension is abolished by high-dose aspirin or picotamide, which is a dual TXA_2_ synthase and TP receptor antagonist [25]. This is also consistent with the pathophysiology of preeclampsia, in that the beneficial effect of low-dose aspirin is primarily attributed to a reduction in TXA_2_ [6], and hence, reduced TP receptor activation. Further, previous studies have demonstrated during pathological situations such as hypertension, obesity and diabetes, that PGI_2_ elicits vasoconstriction via stimulation of TP receptors [24,26–31]. A caveat of this is that this phenomenon is mainly studied in aortic vessels, which may explain why we did not detect any changes in the vasodilator response to ACh in the presence or absence of NO synthase inhibition with l-NAME or calcium channel blockade with apamin and TRAM 34 in our experiments which were conducted in segments of isolated iliac vessels. Here, we must also acknowledge that a limitation of our study is that the vascular function experiments were performed iliac vessels which are not a resistance vessel. Detailed *in vivo* and *in vitro* studies are required to dissect out the contribution of the various prostanoid receptors to the effects of PGI_2_ during angiogenesis-inhibitor therapy.

In the present study, low- and high-dose aspirin blunted the pressor response to sunitinib by a similar extent, suggesting that COX-1-dependent PGI_2_ generation contributes to angiogenesis inhibitor-induced hypertension. This is in-line with previous studies in the spontaneously hypertensive rat demonstrating a key role for COX-1-dependent PGI_2_ generation in endothelium-dependent contractions, such that PGI_2_ acts an endothelium-derived contracting factor (EDCF). Yet, we did not observe a reduction in PGI_2_ in the sunitinib group that was co-treated with low-dose aspirin. This may be explained by the fact that blood samples were collected at the end of day 8 of treatment, i.e., 24 h after the last dose of aspirin was administered. Previous studies have demonstrated that recovery from low-dose aspirin-induced inhibition of endothelial PGI_2_ formation is complete 24 h after the last dose, suggesting that COX-1 is rapidly turned over in the endothelium, at least in humans [32]. Similarly, following treatment with aspirin in cultured vascular endothelial cells from humans, rats and bovine, recovery of PGI_2_ production took up to 24 h [33]. However, in this study there was no discrimination between COX-1 and COX-2-dependent PGI_2_ generation. The 30% reduction in circulating PGI_2_ observed in sunitinib group that was co-treated with high-dose aspirin likely reflects a reduction in COX-2 dependent PGI_2_ generation. Further, while circulating TXA_2_ and PGF_2α_, levels were unchanged during treatment with sunitinib alone, co-treatment with low- and high-dose aspirin significantly reduced these prostanoids. Thus, it is likely that reductions in these prostanoids contributed to the attenuated pressor response to sunitinib during co-treatment with low- and high-dose aspirin. As such, we postulate that COX inhibition (such as that achieved with aspirin) will exert greater protective effects during angiogenesis-inhibitor therapy than targeting a specific prostanoid or prostanoid receptor. Further studies are warranted to test this hypothesis.

The up-regulation in vascular O_2_^−^ during treatment with sunitinib alone most likely contributed to the effects of COX-1-dependent PGI_2_ generation on vascular tone. It is known that O_2_^−^ facilitates EDCF-mediated contractions, either directly by acting as an EDCF or indirectly by reducing the bioavailability of NO and stimulating COX-dependent prostanoid generation. In canine basilar artery, endothelium-dependent contractions are prevented by SOD, which converts O_2_^−^ into H_2_O_2_, but not by catalysis which scavenge H_2_O_2_ [34]. Similarly, in spontaneously hypertensive rats, reactive oxygen species (ROS)-dependent EDCF-mediated contractions were prevented by SOD, indomethacin (a non-specific COX inhibitor). These observations suggest an important role for O_2_^−^ in EDCF-mediated contractions. However, we previously demonstrated that the SOD mimetic tempol does not alter the pressor response to sunitinib in rats [4]. Thus, the increase in O_2_^−^ seems to be secondary to the rise in ET-1 and PGI_2_ during treatment with sunitinib. Consistent with this hypothesis, it has been shown that ET-1 up-regulates COX-1 and COX-2 via ROS, with increased aortic Nox activity and a reduction in SOD [35]. Further, in the present study, treatment with both high- and low-dose prevented the sunitinib-induced increase in O_2_^−^, suggesting that the up-regulation in O_2_^−^ during angiogenesis-inhibitor therapy is COX-dependent.

Activation of the ET likely plays a key role in the activation of the prostanoid biosynthesis pathway during angiogenesis-inhibitor therapy [16]. In preclinical studies, we, and others, have demonstrated that ET_A/B_ or ET_A_ receptor blockade blunts angiogenesis inhibitor-induced hypertension and proteinuria [4,16,36,37]. Importantly, we recently demonstrated that ET_A_ receptor blockade lowers PGI_2_ to a similar extent as dual COX inhibition during sunitinib-induced hypertension [16]. ET-1 leads to an up-regulation of both COX-1 and COX-2, with intrarenal or systemic administration of ET-1 augmenting plasma PGI_2_ [31]. In addition to ET-1 [38], other factors such as inflammatory mediators e.g., nuclear factor-κB (NF-κB) [39], hyperosmolality [40] and hypoxia [41] are key drivers for an up-regulation in COX-2. Angiogenesis inhibitors are known to cause hypoxia-induced up-regulation of COX-2, with concomitant COX-2 inhibition resulting in synergistic effects with angiogenesis-inhibitor therapy as PGE2 is an angiogenic factor [42]. Thus, COX-2, and hence PGI_2_, may be up-regulated during angiogenesis-inhibitor therapy due to hypoxia (independent of ET-1) and/or the up-regulation in ET-1.

In the present study, albuminuria was increased during sunitinib treatment, yet we did not detect a significant difference in the renal mRNA expression of the podocyte-specific molecules, podocin or nephrin. This is consistent with clinical data which reported that the cumulative dose of bevacizumab is positively correlated with albuminuria rather than the urinary mRNA expression nephrin, podocin and VEGF-A [43]. Also, transmission electron micrographs demonstrated that sunitinib induced a loss of endothelial fenestrations and mild endothelial activation, which can be indicative of glomerular endothelial injury [44]. Importantly, high-dose aspirin, but not low-dose aspirin, completely prevented the development of sunitinib-induced albuminuria and this effect was associated with a reduction in urinary PGI_2_ excretion. The latter finding is in contrast with Robinson et al., who did not observe a change in urinary PGI_2_ after angiogenesis-inhibitor therapy in patients [45]. Nevertheless, our data suggest that angiogenesis inhibitor-induced renal injury is mediated, at least in part, via COX-2-dependent PGI_2_ generation. Consistent with the rise in circulating PGI_2_, we postulate that the massive up-regulation of renal PGI_2_ production during angiogenesis-inhibitor therapy most likely occurs as a protective mechanism. PGI_2_ plays a critical role in the maintenance of renal blood flow and glomerular filtration rate when actual or effective circulating volume is decreased by blunting vasoconstriction of the pre-glomerular afferent arteriole [46]. Thus, the up-regulation in PGI_2_ may occur to offset renal NO deficiency [4,47] and the shift in the pressure–natriuresis curve [13,47] during angiogenesis-inhibitor therapy. Further studies are necessary to delineate the effect of PGI_2_ on renal autoregulation and pressure-natriuresis during angiogenesis-inhibitor therapy and whether blocking COX-2 to prevent the kidney injury associated with these drugs will adversely affect renal function.

## Conclusion

The present study provides a rationale for the use of COX inhibitors, particularly COX-2 inhibition, for the prevention of angiogenesis inhibitor-induced hypertension and renal injury. Theoretically, COX inhibition is an attractive option in patients receiving angiogenesis inhibitor therapy as other benefits including a reduction in thrombotic tendency which is COX-1/TP receptor mediated [21,48] and the inhibition of tumour growth and prevention of resistance to angiogenesis-inhibitor therapy, which are both linked to COX-2 [49–53], are likely to be present as well. Further studies are warranted to determine the contribution of the prostanoid pathway to angiogenesis inhibitor-induced hypertension and renal injury and the potential of targeting this pathway as novel strategy to prevent these unwanted effects.

## Clinical perspectives

- **Background as to why the study was undertaken:** Understanding the mechanisms underlying angiogenesis inhibitor-induced hypertension and nephropathy is integral if we are to develop preventative strategies to protect cardiovascular health and allow cancer patients to remain on this life-saving treatment.
- **A brief summary of the results:** Our data demonstrate that PGI_2_ is paradoxically up-regulated during angiogenesis-inhibitor therapy. Low-dose aspirin (COX-1 inhibition) and high-dose aspirin (dual COX inhibition) blunted angiogenesis inhibitor-induced hypertension to a similar extent, whereas only high-dose aspirin prevented albuminuria.
- **The potential significance to human health and disease:** Our results uncover a novel role for PGI_2_ during angiogenesis-inhibitor therapy and suggest that targeting the prostanoid pathway may be a novel strategy to prevent angiogenesis inhibitor-induced hypertension and kidney damage.

## Supplementary Material

Supplementary Figures S1-S3

## Data Availability

All supporting data are included within the article.

## Open Access

Open access for this article was enabled by the participation of Monash University in an all-inclusive *Read & Publish* agreement with Portland Press and the Biochemical Society under a transformative agreement with CAUL.

## Abbreviations

ACh: acetylcholine
COX: cyclo-oxygenase
EDCF: endothelium-derived contracting factor
ET: endothelin
*Hprt* *1*: hypoxanthine phosphoribosyltrasferase-1
H_2_O_2_: hydrogen peroxide
KIM-1: kidney injury molecule-1
l-NAME: l-ω-Nitro-l-arginine methyl ester hydrochloride
NGAL: neutrophil gelatinase-associated lipocalin
Nox: NADPH oxidase
PAS: Periodic Acid–Schiff
PGE: prostaglandin E
PGE-M: prostaglandin E metabolite
PGI_2_: prostacyclin
ROS: reactive oxygen species
sFlt-1: soluble Fms-like tyrosine kinase 1
SU: sunitinib
TBS: Tris-buffered saline
TKI: tyrosine kinase inhibitor
TXA_2_: thromboxane
VEGF: vascular endothelial growth factor
VEGFR: VEGF receptor
WKY: Wistar Kyoto
