# Supplementary material for: Differential effects of cyclo-oxygenase 1 and 2 inhibition on angiogenesis inhibitor-induced hypertension and kidney damage
Source: Clin Sci (Lond). 2022 May 10;136(9):675–94. doi: 10.1042/CS20220182 (PMC9093150; doi:10.1042/CS20220182)

Supplemental Figs

Supplemental Figure 1. Representative light microscopy images of kidney sections stained with PAS following treatment with (**A)** vehicle, **(B)** sunitinib (14 mg/kg/day; SU) alone or during co-treatment with **(C)** low-dose aspirin (COX-1 inhibition, 5 mg/kg/day; SU+low-dose A) or **(D)** high-dose aspirin (dual COX-1 and COX-2 inhibition, 100 mg/kg/day; SU+high-dose A). Magnification 40x.

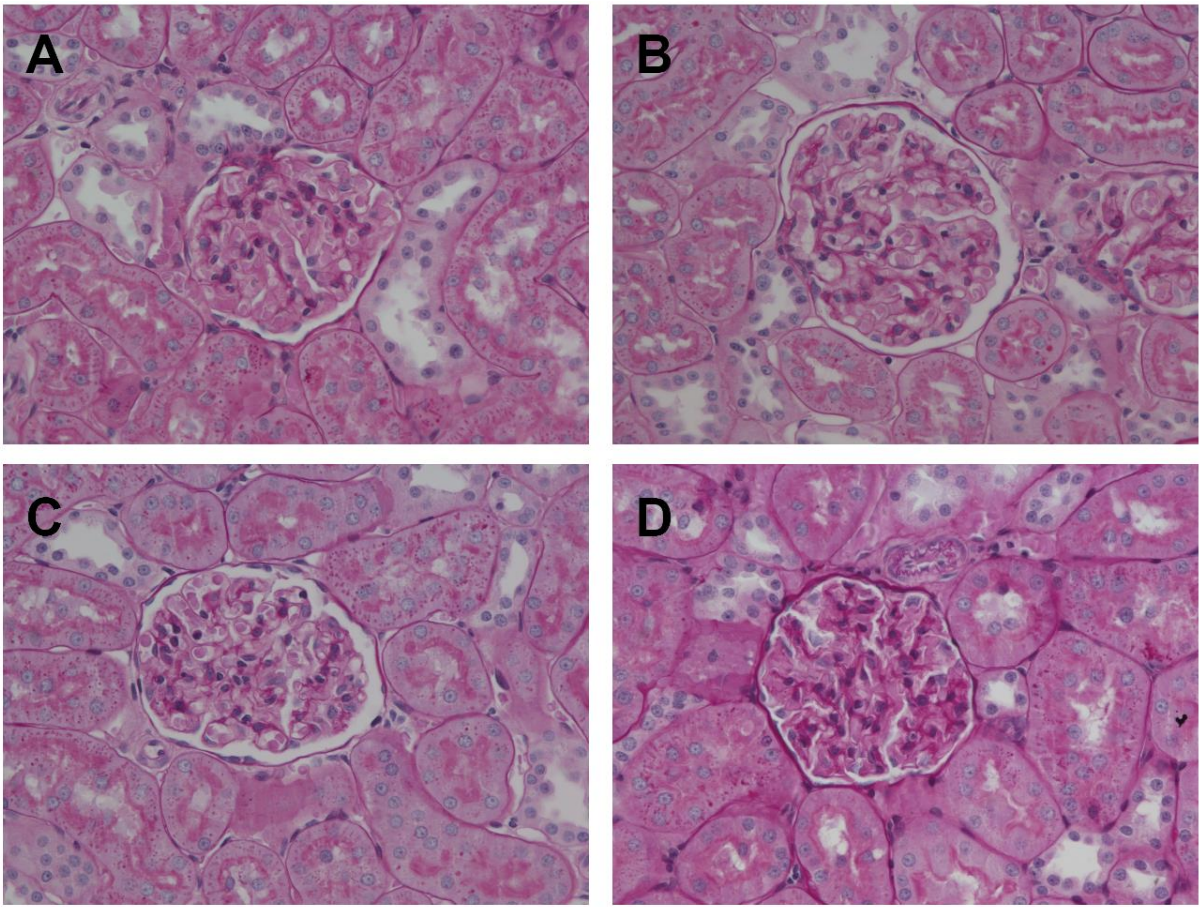

Supplemental Figure 2. Transmission electron micrographs of kidney sections from WKY rats treated with (**A-B)** vehicle or **(C-D)** sunitinib (14 mg/kg/day; SU). (A-B) normal capillary lumina (number sign) and endothelial cells with preserved fenestration (arrows) in vehicle treated rats. (C-D) endothelial swelling, indicative of endothelial activation (asterix) and mild loss of endothelial fenestrations (arrowheads). RBC indicates red blood cell.

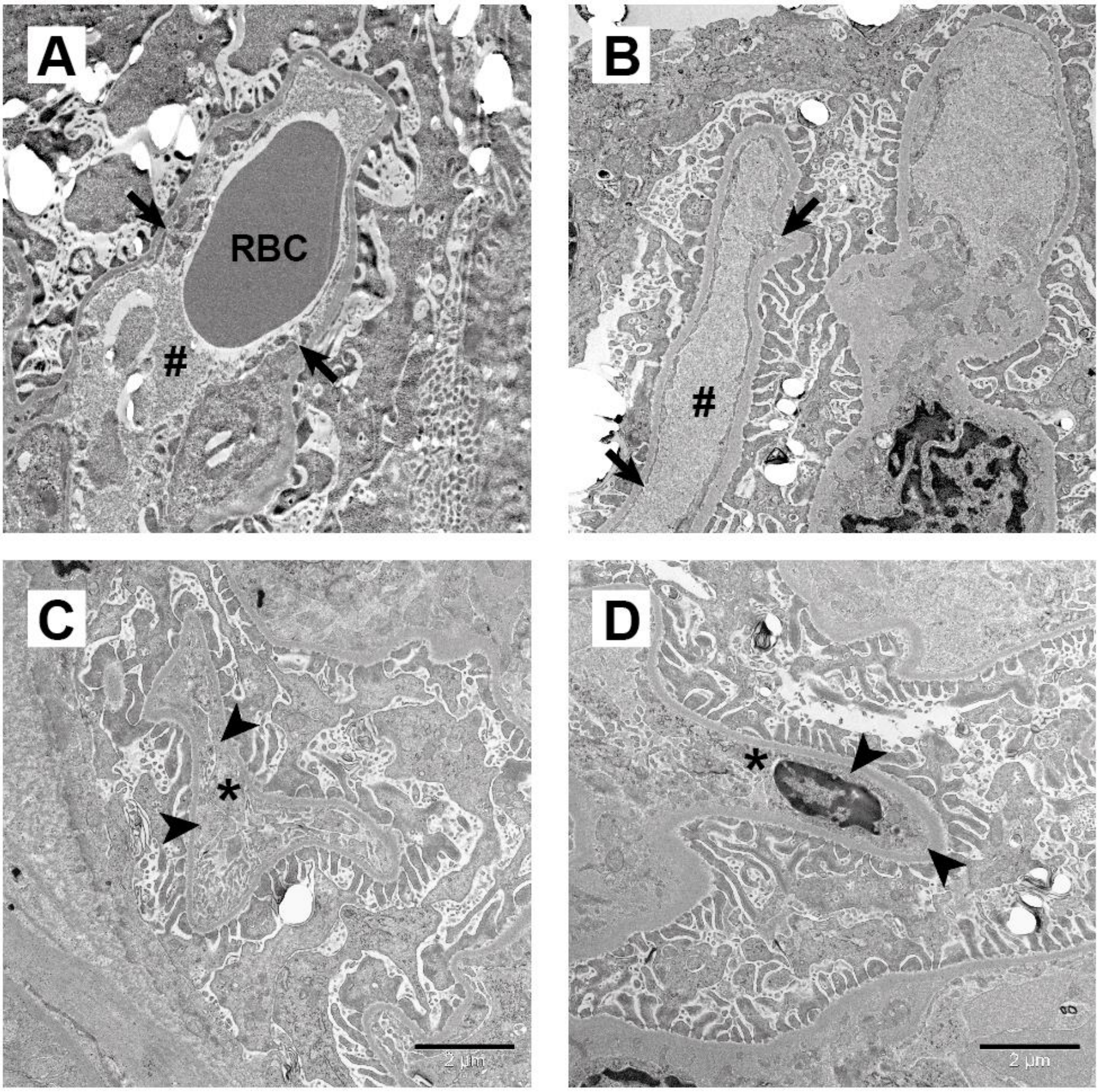

Supplemental Figure 3. Renal mRNA expression of **(A)** vascular endothelial growth factor (VEGF), **(B)** cyclooxygenase (COX)-1, **(C)** COX-2, **(D)** PGI_2 synthase and **(E)** TXA_2 synthase following treatment with vehicle, sunitinib (14 mg/kg/day; SU) alone or during co-treatment with low-dose aspirin (COX-1 inhibition, 5 mg/kg/day; SU+low-dose A) or high-dose aspirin (dual COX-1 and COX-2 inhibition, 100 mg/kg/day; SU+high-dose A). Renal mRNA expression is normalized to the internal housekeeping gene hypoxanthine phosphoribosyltrasferase-1 (Hprt1) and are expressed relative to the vehicle treated group. Data are presented as mean ± SEM (n=5-7/group). Data were analyzed using a one-way ANOVA.

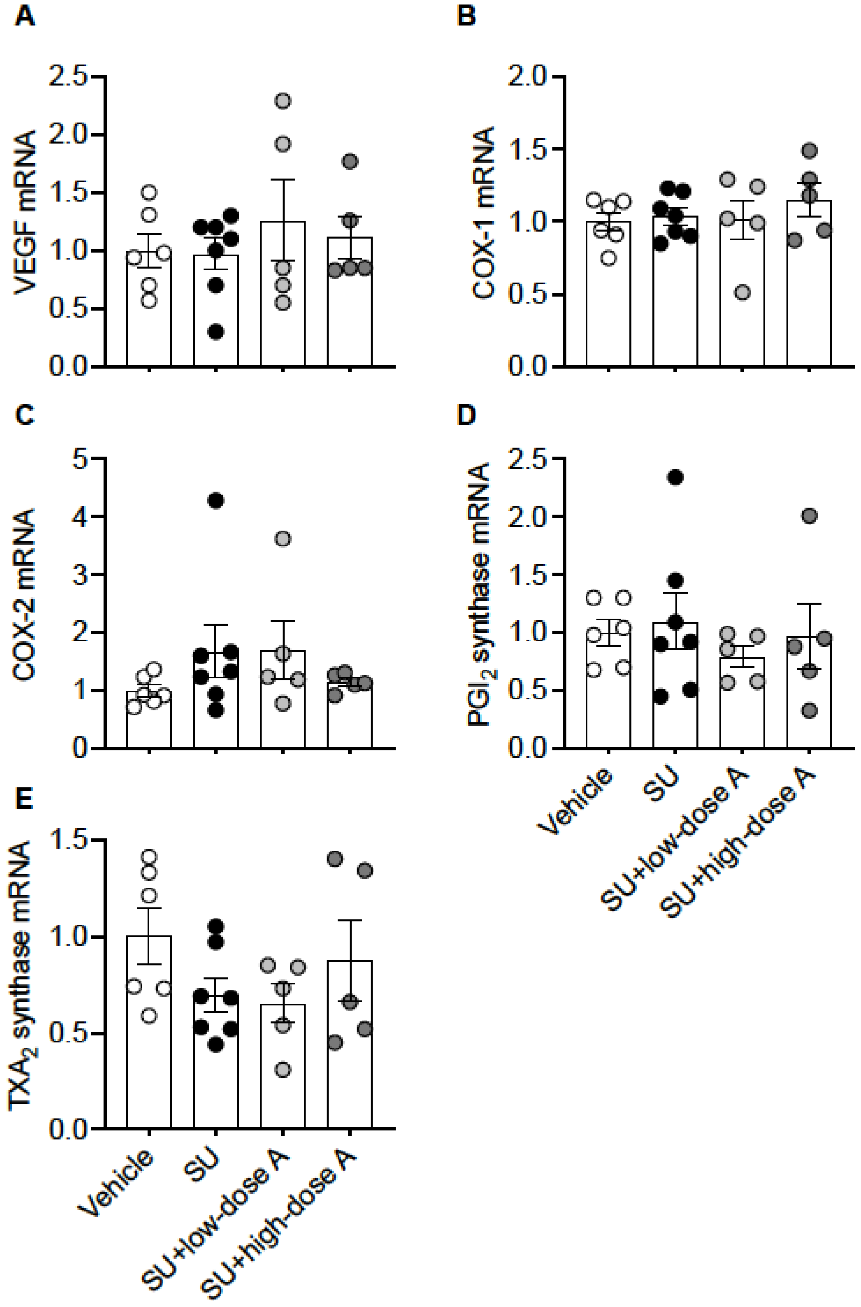

Supplement: Supplementary Figures S1-S3 [file CS-2022-0182_supp.pdf]
